# Fabrication and biological evaluation of chitosan coated hyaluronic acid-docetaxel conjugate nanoparticles in CD44^+^ cancer cells

**DOI:** 10.1186/s40199-016-0160-y

**Published:** 2016-07-29

**Authors:** Nazanin Shabani Ravari, Navid Goodarzi, Farhad Alvandifar, Mohsen Amini, Effat Souri, Mohammad Reza Khoshayand, Zahra Hadavand Mirzaie, Fatemeh Atyabi, Rassoul Dinarvand

**Affiliations:** 1 Nanotechnology Research Centre, Faculty of Pharmacy, Tehran University of Medical Sciences, Tehran, 1417614411 Iran; 2 Nanomedicine and Biomaterial Lab, Department of Pharmaceutics, Faculty of Pharmacy, Tehran University of Medical Sciences, Tehran, Iran; 3 Department of Medicinal Chemistry, Faculty of Pharmacy, Tehran University of Medical Sciences, Tehran, Iran; 4 Department of Drug and Food Control, Faculty of Pharmacy and Pharmaceutical Quality Assurance Research Center, Tehran University of Medical Sciences, Tehran, Iran

**Keywords:** Glyconanoparticles, Nanomedicine, Polyelectrolye Complex, Macromolecular Drug Delivery, Polysaccharides

## Abstract

**Background:**

Hyaluronic acid (HA) has been used for target-specific drug delivery because of strong affinity to CD44, a marker in which overexpressed in cancer cells and cancer stem cells. Conjugation of HA to the cytotoxic agents via active targeting can improve efficacy, biodistribution, and water solubility. To be able to benefit from passive targeting as well, a nanoparticulate system by counter ion using a polycation like chitosan may lead to a perfect delivery system.

**Methods:**

Water soluble Hyaluronic acid-Docetaxel (HA-DTX) conjugate was prepared and used to formulate chitosan-coated HA-DTX nanoparticles by polyelectrolyte complex (PEC) method and optimized using Box-Behnken design. Biological evaluation of nanoparticles was done in CD44+ cancer cells.

**Results and discussion:**

Biological evaluation of optimized formula showed IC50 of nanoparticles for 4 T1 and MCF-7 cell lines were 45.34 μM and 354.25 μM against 233.8 μM and 625.9 μM for DTX, respectively with increased cellular uptake showed by inverted confocal microscope.

**Conclusion:**

Chitosan-coated HA-DTX nanoparticles were more effective against CD44+ cells than free DTX.

**Graphical abstract:**

Chitosan coated hyaluronic acid-docetaxel conjugate nanoparticles fabricated and evaluated in CD44+ cancer cells
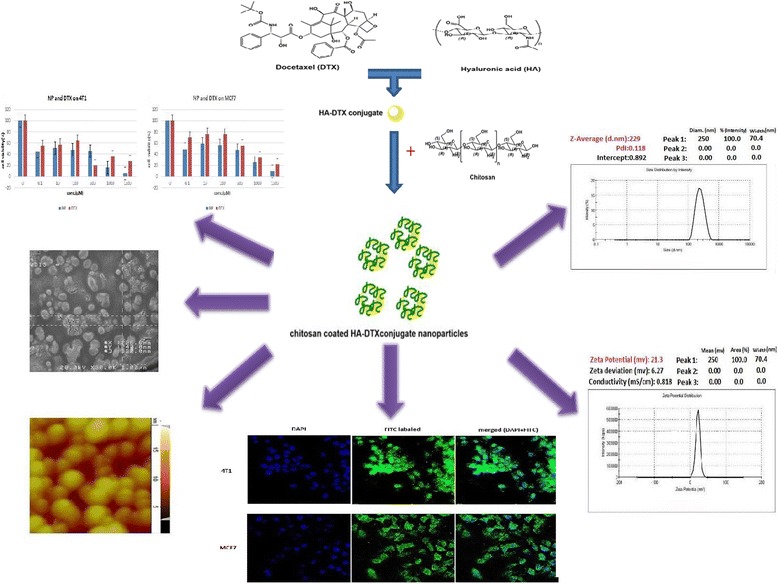

## Background

Most of the anticancer drug products have systemic toxicity because of the wide uncontrolled distribution in the body. Besides, their lack of tumor localization and short half-lives are considerable obstacle facing effective cancer chemotherapy. Development of nanoparticulate drug delivery systems and polymer-drug conjugates of low molecular weight cytotoxic drug molecules to macromolecular carriers are effective ways to address these problems by enhanced permeation and retention (EPR) effect [1, 2]. In addition, conjugation of cytotoxic drugs to hydrophilic macromolecules can increase the water solubility of insoluble drugs such as docetaxel (DTX) and will enhance their biodistribution and therapeutic efficacy [3, 4].

DTX is an anticancer agent belongs to the Taxanes family and is a semi-synthetic derivative from the *Taxus brevifolia* [5] DTX shows its cytotoxic effect by inhibiting the depolymerization of microtubules and M-phase cell arrest [6]. The conventional formulations of DTX in drug market suffering from low solubility of the active pharmaceutical ingredient which has been resolved using tween 80 as surfactant. This issue led to some complications in the clinic to control formulation-related adverse drug reactions [7]. Due to the importance of this drug molecule in chemotherapy protocols, a lot of efforts dedicated to address novel formulations of DTX. In one of these approaches water-soluble macromolecular drug conjugates has been proposed to prepare tween-free formulations along with targeted drug delivery such as hyaluronic acid-docetaxel (HA-DTX) conjugates. In our previous report [4], although prepared conjugate had better solubility profile but the efficacy did not benefit from its polysaccharide CD44-targeting properties and heparine like effects of hyaluronic acid in blood circulation. Therefore this report is an attempt to add passive targeting enhanced permeation and retention of nanoparticulate system while keeping hyaluronate moiety. For this purpose, the water soluble conjugate became coated with chitosan by polyelectrolyte complex (PEC) method to prepare a nanoparticulate drug delivery system to improve pharmacokinetic features and efficacy.

For this purpose, hyaluronic acid (HA) as one of the common polysaccharides carriers has been used for conjugation of low molecular weight cytotoxic drugs such as DTX. HA is biocompatible, biodegradable and non-immunogenic [8]. The most considerable advantage of HA is its strong affinity for CD44, a cell surface protein which is overexpressed in many cancer cells and cancer stem cells [9, 10]. CD44 is a specific biological receptor for HA [11, 12]. HA could have an enhanced attachment and uptake into malignant cells with metastatic activities [13] and has been used for target-specific drug delivery [14, 15]. We hypothesized using HA as carrier and targeting moiety simultaneously may reverse the multiple drug resistance of cancer stem cells via affecting CD44 and regarding physical correlation of P-gp and these markers [16]. In this regard, HA-DTX conjugates has been prepared and evaluated which showed suitable efficacy and safety profile. This research reports optimized polyelectrolyte complex nanoparticles using HA-DTX as cationic part.

Polyelectrolyte complexes which were prepared by electrostatic interaction between unlike charged poly-ions have received substantial attention in drug delivery systems. The synthesis of PEC nanoparticles is simple and can be easily carried out under mild conditions without using toxic organic solvents or chemical cross-linkers [17]. In the present study, chitosan as a N-deacetylated derivative of chitin has been used extensively as a biocompatible polysaccharide [18] with a cationic nature that can be protonated in weak acidic environment [19], and therefore it can improve the bioavailability of DTX [20].

Achieving optimization of the nanoparticle preparation could be performed by classical method of changing one variable at a time while others have been remained constant. However this method needs lots of series of experiments and time. Moreover in this approach the possible interaction between independent factors will not be observed. Therefore, the fine optimized formulation will not be achieved. Design-of-experiment (DoE) method has been used in pharmaceutical studies to solve this problem. Optimization by response surface methodology including Box-Behnken method is considered as the major application of DoE [21, 22].

In the present study, the water soluble conjugated HA-DTX was synthesized. Then, chitosan coated HA-DTX nanoparticles were prepared by PEC method considering the anionic structure of HA-DTX and cationic chitosan. Box-Behnken statistical design using a response surface methodology has been employed to obtain the optimized condition in terms of particle size, size distribution, drug loading and zeta potential. For determination of efficacy of optimized chitosan coated HA-DTX conjugate nanoparticles, 3-(4,5-dimethylthiazol-2-yl)-2,5-diphenyltetrazolium bromide (MTT) assay was performed on MCF-7, human cancer cell line, and 4 T1 mouse breast cancer cell line. MCF-7 and 4 T1 cell lines were also used for cell uptake study.

### Materials and methods

#### Materials

Sodium hyaluronate (MW 25 kDa) was purchased from GuangLong (Shandong, China). Anhydrous DTX was purchased from Jiangsu Yew (Jiangsu, China). Chitosan (MW 50 kDa, (Primex, Karmoy, Norway), 1-Ethyl–3-[3-(dimethylamino)-propyl] carbodiimide (EDC), N-hydroxy succinimide (NHS), 4’,6-diamidino-2-phenylindole (DAPI) and triethylamine were purchased from Sigma Aldrich (Seelze, Germany). MTT dye was from Merck (Darmstadt, Germany). Dulbecco's Modified Eagle Medium (DMEM) with high glucose, RPMI 1640, FBS (Fetal Bovine Serum), trypsin, penicillin and streptomycin were purchased from Biosera (Vienna, Austria). Ultra-purified water was used throughout the analysis and all other chemicals were of analytical grade. 4 T1 and MCF-7 cell lines were obtained from National Cell Bank of Iran (Pasteur Institute of Iran, Tehran, Iran).

## Methods

### Synthesis of hyaluronic acid-docetaxel (HA-DTX) conjugates

At first for preparing desalted HA, HA (MW 25 kDa) (1 g) was dissolved in 100 mL of deionized water, then the solution was dialyzed (MWCO 12 kDa) in deionized water for 24 h and lyophilized [23].

Desalted-HA (400 mg) was dissolved in 80 mL of deionized water. Then EDC and NHS were added to the solution in 11 and 10 molar ratios of carboxyl groups of HA, respectively. The mixture was stirred at 40 °C for 3 h. Then 80 mL dimethylformamide (DMF) containing 400 mg of DTX and 20 mL of triethylamine were added. After 24 h the mixture was refluxed at 70 °C and after cooling to room temperature was transferred to pretreat dialysis tubing (MWCO 3500 kDa). The mixture was purified by dialysis against water-acetone solution (50:50, v/v) for 1 h, water-acetone (75:25, v/v) for 1 h and water for 1 h, respectively. The obtained HA-DTX conjugate is water soluble. To eliminate the remained DTX, the mixture was transferred to a separating funnel and extracted three times with dichloromethane. The aqueous phase was lyophilized. Chemical integrity of the resulted product was checked by ^1^H-NMR (Bruker AC 500 Spectrophotometer, Germany) and fourier transform infra-red (FTIR) spectroscopy (Nicolet Magna-FTIR 550 Spectrometer, WI, USA). The concentration of DTX was measured by UV Spectrophotometry (UV-visible Spectrophotometer, 160A, SHIMADZU, Japan) at 229 nm versus a suitable blank solution containing the appropriate concentration of HA.

### Preparation of chitosan coated HA-DTX conjugate nanoparticle

Chitosan coated HA-DTX conjugate nanoparticles were prepared by PEC method, considering the anionic structure of HA-DTX conjugates and cationic chitosan [24]. As a representative example, 1 mL of aqueous solution of HA-DTX (4.25 mg/mL) was added slowly to 1 mL of dilute chitosan solution (0.250 mg/mL of 1 % acetic acid) in 1 min while stirring at 370 rpm at room temperature. It should be mentioned that during the screening and optimization procedure, various factors including ratios of HA-DTX conjugate to chitosan, stirring rate and temperature were evaluated.

### DTX determination in the conjugates and nanoparticles

The concentration of DTX in conjugate was measured by UV spectrophotometry. Determination of DTX in prepared nanoparticles was performed by UV absorbance at 229 nm to determine drug loading and entrapment efficiency. The final solution of chitosan coated HA-DTX nanoparticles were transferred to microtubes and centrifuged by an ultracentrifuge (Optima MAX-XP Ultracentrifuge, Beckman Coulter, USA) at 22000 rpm (150700 g) for 20 min at 10 °C. After collecting the supernatant, the free remaining non-conjugated DTX in the reaction medium was measured by UV spectrophotometry. The encapsulated efficiency and DTX loading in nanoparticles were calculated, applying the following equations:

$$ \begin{array}{l} Entrapment\kern0.5em  efficiency\kern0.5em \left(\%\right)=\frac{Total\kern0.5em  amount\kern0.5em  of\kern0.5em DTX- Amount\kern0.5em  of\kern0.5em DTX\kern0.5em  in\kern0.5em  supernatant}{Total\kern0.5em  amount\kern0.5em  ofDTX}\times 100\hfill \\ {} Drug\kern0.5em  loading\kern0.5em \left(\%\right)=\frac{Weight\kern0.5em  of\kern0.5em  drug\kern0.5em  found\kern0.5em  loaded}{Weight\kern0.5em  of\kern0.5em  nanoparticle}\times 100\hfill \end{array} $$

### Fourier transform infra-red spectroscopy of conjugates

Freeze-dried conjugated HA-DTX, HA, DTX were analyzed by FTIR Spectrometer. The data was achieved in the range of 400–4000 cm^−1^ for each sample. The FTIR spectra of conjugated HA-DTX were compared with pure substances.

### Experimental design studies

Box-Behnken statistical design which is a response surface methodology has been employed in the present study. In this study the effect of three quantitative independent variables consisting of stirring rate (rpm), ratio of HA-DTX conjugate to chitosan and temperature (°C) were investigated on dependent variables and responses, including particle size (nm), zeta potential (mv), polydispersity index (PdI) and DTX loading in nanoparticles with Design Expert software (V. 7.0.0, Stat-Ease Inc., Minneapolis, USA). Dependent and independent variables were elucidated based on the preliminary studies which are shown in Table 1.

**Table 1:** Variables used in Box-Behnken experimental design

Independent factors	Factor level			Dependent factors
Numeric Factors	−1	0	1	Particle size (nm)
Stirring rate (rpm)	300	800	1300	Zeta potential (mv)
Ratio of HA-DTX conjugate to chitosan	13	17	21	Polydispersity index (PdI)
Temperature (°C)	0	25	50	Drug content or DTX loading (%)

The aim of the design was to achieve to the optimum formulation, with both minimum size and maximum loading. The PdI factor should be at possible lowest level and zeta potential should be appropriate to have stable nanoparticles. Obtained responses from three optimized formulations were compared with the suggested experimental responses to evaluate the precision of model.

### Characterization of the nanoparticles

The size and zeta potential of the nanoparticles were determined using a Zetasizer Nano ZS Analyzer (Malvern Instruments, UK) with a He-Ne laser beam at wavelength of 633 nm at 25 °C.

Surface morphology of the nanoparticles observed using Scanning Electron Microscopy (SEM) (Philips Xl30, The Netherlands) and Atomic Force Microscopy (AFM) (dualscope™ DS 95-200/50, Denmark) microscopy. For AFM evaluation, one drop of nanoparticle suspension was dried on the surface of clean silicon wafer at room temperature. AFM study was performed with 20 μm scanner in tapping mode. For SEM imaging, dried nanoparticles were gently coated by gold layer with a sputter coater and evaluated at 30 kV using a 6300 field emission scanning electron microscope.

Differential scanning calorimetry (DSC) was performed using Mettler-Toledo DSC822^e^ (Greifensec, Switzerland) and data acquisition and analysis was carried out by a software package of STAR^e^ 9.01. The system was conducted by using 8 mg of sample, deposited in 40 μL aluminum pans and hermetically sealed, under a nitrogen gas dynamic flow at a scanning heating rate of 10 °C/min over a range of 20 °C to 300 °C. Empty hermetically sealed aluminum pan was used as a control.

### Cytotoxicity evaluation of nanoparticles

For cytotoxicity study of nanoparticles, MTT assay was performed on MCF-7, human breast cancer cell line, and 4 T1 mouse breast cancer cell line [25]. Cell culture medium was DMEM with 10 % FBS and 5 % penicillin-streptomycin. Cells maintained at 37 °C and humidified environment with 5 % CO_2_. Cells were seeded into 96-well plate separately at a seeding density of 5000 cell/well. After 24 h incubation, various concentrations of free DTX and nanoparticles (0.1, 10, 100, 500, 1000, and 1500 μM) (based on DTX equivalent concentration) were used as treatments and incubated for 48 h. Then 50 μL MTT (1 mg/mL) solution in PBS was added to each well and incubated for 4 h. Formazan precipitates dissolved by 150 μM dimethyl sulfoxide (DMSO). The absorption was measured at 570 nm and reference well at 620 nm by ELISA reader [26]. Cell viability was calculated by the following equation where OD is optical density:

$$ \mathrm{Cell}\ \mathrm{viability}\% = \left({\mathrm{OD}}_{\mathrm{test}\ \mathrm{well}}/\ {\mathrm{OD}}_{\mathrm{reference}\ \mathrm{well}}\right)\ \mathrm{x}\ 100 $$

### Preparation of fluorescent-labeled HA

To prepare fluorescent-labeled HA conjugate, HA was labeled with fluorescamine. One hundred mg of HA was dissolved in 10 mL water and 61.5 mg of EDC and 45.5 mg of NHS were added. The mixture was stirred for 3 h and then 45.84 mg fluorescamine was added and stirred for 24 h. The reaction flask was protected from light by covering with an aluminum foil. After 24 h the mixture was purified by dialysis (MWCO 3500) against deionized water and finally the product was freeze-dried. Fluorescent-labeled nanoparticles were also prepared by fluorescent-labeled HA conjugate instead of HA-DTX conjugate and the nanoparticle preparation method was the same as the optimized method used for normal nanoparticles.

### Cell uptake studies

In order to study the cellular uptake of the nanoparticles, 4 T1 and MCF-7 cell lines were seeded at 1 × 10^5^ cell/well in a cover glass and incubated at 37 °C with 5 % CO_2_ atmosphere for 24 h. After complete adhesion, the medium was carefully removed and replaced with fresh medium containing fluorescent-labeled nanoparticles and incubated for 2 h. In this stage, the medium containing drug was removed and cells were washed 4 times with PBS and fixed with formaldehyde 4 % for 4 min. Nuclear coloring was performed with DAPI (0.5 mg/mL) in 5 min and then cells were washed 4 times with PBS using inverted confocal microscope (Nikon ECLIPSE Ti, Tokyo, Japan) cell images were taken [27].

### Statistical analysis

SPSS 20.0 statistical software and one-way analysis of variance (ANOVA) were used to assess the data groups. All the results were evaluated as mean ± standard deviation (SD). Significance difference of *p* < 0.05 was accepted.

## Results and discussion

### Synthesis and characterization of water-soluble HA-DTX direct conjugate

**Fig. 1:**
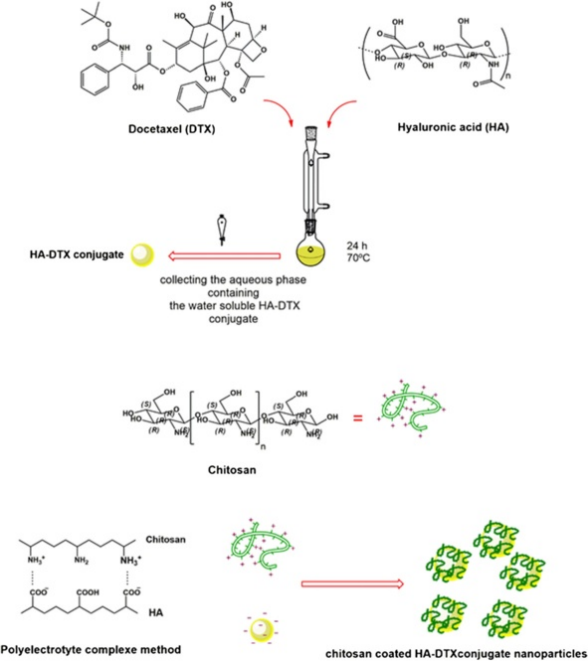
Schematic representation of hyaluronic acid-docetaxel (HA-DTX) conjugate and chitosan coated HA-DTX nanoparticles preparation

**Fig. 2:**
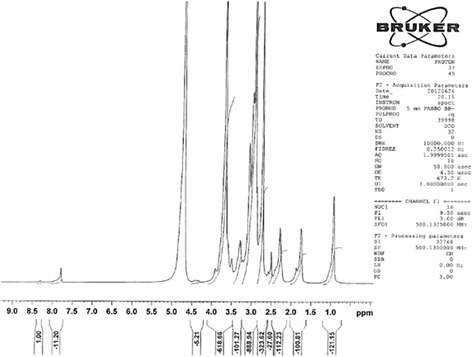
Formation of hyaluronic acid-docetaxel (HA-DTX) conjugate was confirmed by the presence of aromatic protons in ^1^H-NMR spectra

**Fig. 3:**
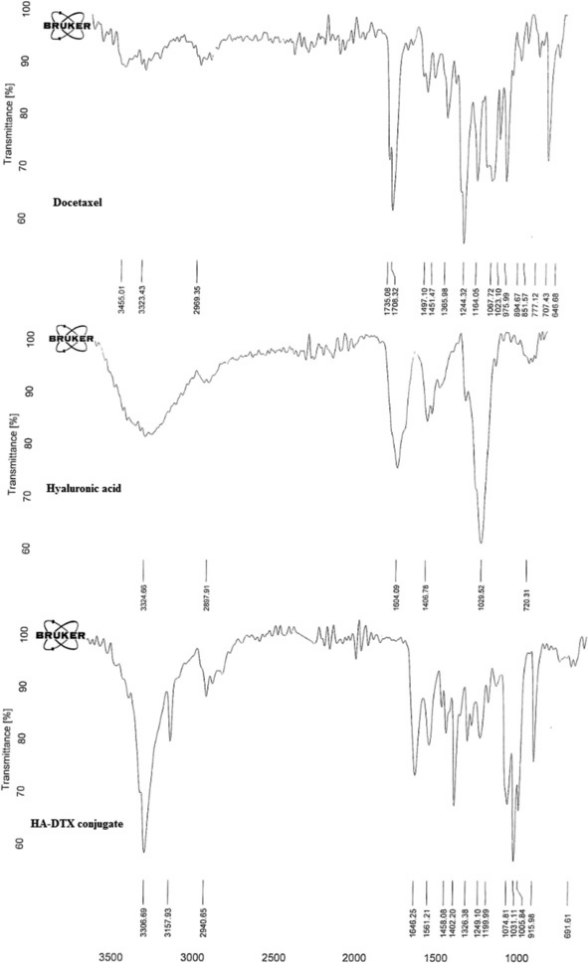
FTIR spectrum of (**a**) docetaxel (DTX); **b** hyaluronic acid (HA); **c** hyaluronic acid-docetaxel (HA-DTX) conjugate

Indirect HA-DTX conjugate was synthetized previously in our group. Conjugation of DTX in these studies needs preparation of succinyl DTX [4, 28]. The direct formation of conjugated HA-DTX has been reported for the first time with fewer procedure steps (Fig. 1). An esteric bound formed between 2´-OH of DTX and COOH of HA [29]. Formation of conjugated HA-DTX was confirmed by the presence of aromatic protons in ^1^H-NMR spectra (Fig. 2). FTIR of freeze-dried HA-DTX conjugate, pure HA, pure DTX were also obtained (Fig. 3). DTX had a specific peak in 1242 cm^−1^ which had no interaction with HA specific peak. This peak has been repeated in HA-DTX conjugate spectrum with a little shift to 1249 cm^−1^. Presence of this peak and HA related peaks in conjugate spectrum confirms the formation of HA-DTX conjugate.

The absorption of DTX in conjugate was measured by UV spectroscopy. According to UV absorption, it was shown that 1 mg of HA-DTX conjugate contains 69 μg of DTX.

### Preparation of chitosan coated HA-DTX conjugate nanoparticle

Publications reported several nanoparticular based methods to improve DTX drug delivery to cancer cells (DTX loaded chitosan nanoparticle [30], targeted DTX nanoparticles with different targeting agents like folic acid [2] and albumin nanoparticles of DTX [28]). HA could cause heparin-induced thrombocytopenia because of its nature as a polysaccharide [31]. This event should be considered if the HA-DTX used alone. Coating the HA-DTX conjugate with chitosan may limit the exposure of HA with platelet in blood circulation and reduce thrombocytopenia. Chitosan-coated HA-DTX conjugate nanoparticles were prepared based on these findings.

### Optimization studies

To obtain an optimized formulation of HA-DTX nanoparticles, Box-Behnken statistical design was used. Table 2 shows 17 runs based on Box-Behnken design to analyze the effects of independent variables on dependent variables and the data achieved to find out optimized formulation.

**Table 2:** The effect of independent variables on dependent variables

Run	Independent variables			Dependent variables			
	Stirring rate (rpm)	Ratio of HA-DTX conjugate to chitosan	Temperature (°C)	Particle size (nm)	Zeta potential (mv)	Polydispersity index (PdI)	DTX loading (%)
1	300.00	13.00	25.00	195	21.7	0.128	3.043
2	300.00	21.00	25.00	444	18.6	0.065	3.257
3	1300.00	13.00	25.00	176	22.3	0.129	3.034
4	1300.00	21.00	25.00	269	17.4	0.030	3.235
5	800.00	13.00	0.00	174	22.3	0.120	3.042
6	800.00	21.00	0.00	280	18.0	0.019	3.235
7	800.00	13.00	50.00	182	21.9	0.086	3.048
8	800.00	21.00	50.00	246	19.2	0.019	3.233
9	300.00	17.00	0.00	210	21.3	0.083	3.159
10	1300.00	17.00	0.00	199	19.6	0.033	3.161
11	300.00	17.00	50.00	210	21.3	0.031	3.157
12	1300.00	17.00	50.00	194	24.1	0.070	3.160
13	800.00	17.00	25.00	195	21.3	0.061	3.160
14	800.00	17.00	25.00	191	21.6	0.039	3.160
15	800.00	17.00	25.00	179	22.7	0.064	3.159
16	800.00	17.00	25.00	193	21.1	0.045	3.159
17	800.00	17.00	25.00	197	21.3	0.067	3.159

As a result, 17 runs were needed to achieve the optimized formulation and the second-order polynomial functions explained the relationship between the dependent and the independent variable as following equation:

$$ {\mathrm{Y}}_{1,\ 2,\ 3} = {\mathrm{b}}_0+{\mathrm{b}}_1\mathrm{A}+{\mathrm{b}}_2\mathrm{B}+{\mathrm{b}}_3\mathrm{C}+{\mathrm{b}}_{11}{\mathrm{A}}^2+{\mathrm{b}}_{22}{\mathrm{B}}^2+{\mathrm{B}}_{33}{\mathrm{C}}^2+{\mathrm{b}}_{12}\mathrm{AB}+{\mathrm{b}}_{13}\mathrm{AC}+{\mathrm{b}}_{23}\mathrm{B}\mathrm{C} $$

which A, B and C are independent variables, and Y is the predicted dependent factor, b_0_ is the intercept, b_1_, b_2_, and b_3_ are linear coefficients, b_11_, b_22_, and b_33_ are squared coefficients, and b_12_, b_13_, and b_23_ are the interaction coefficients of equation.

#### Size of nanoparticles

**Fig. 4:**
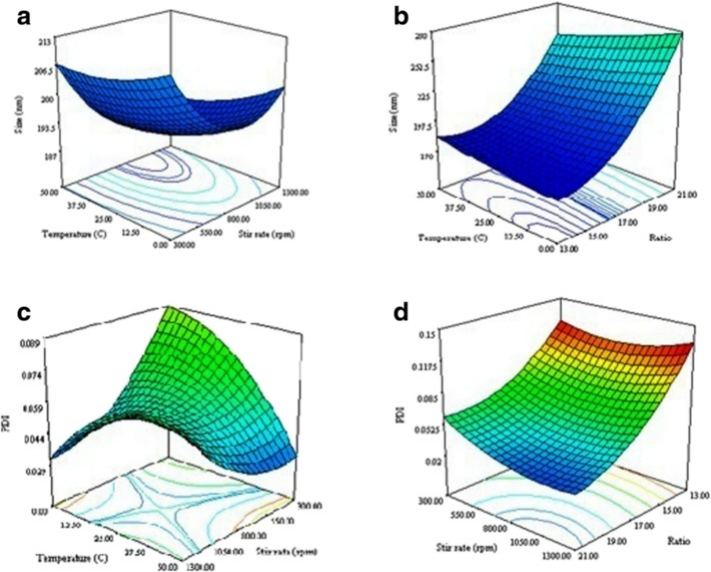
Response surface plots showing the effect of different independent factors on (**a**) (**b**) size of nanoparticles; **c**, **d** polydispersity index

Size is the most important parameter in determining the nanoparticles cellular uptake. Nanoparticles were optimized to achieve minimum size while the PdI kept at minimum and loading maximum. As seen in Fig. 4a in the middle range of stirring rate, size of nanoparticles would be reduced. Higher stirring rate could generate bubble and solution splashing so it could not prepare suitable particles. It seems stirring rate at lower limit do not supply enough energy to product small particles, so enlargement has seen in this rate. Figure 4b represents that by increasing the ratio of HA-DTX to chitosan, the size of nanoparticles would be increased. Temperature had minor effect on the size of nanoparticles.

$$ \mathrm{Particle}\ \mathrm{size}=191+64\mathrm{A}\hbox{-} 27.63\mathrm{B}\hbox{-} 3.87\mathrm{C}+48.62{\mathrm{A}}^2+31.38{\mathrm{B}}^2\hbox{-} 19.13{\mathrm{C}}^2 $$

#### Polydispersity index

Polydispersity index represents the homogeneity of nanoparticles and changs between of 0 to 1 with a desire to be near 0. The less PdI value indicates the more size uniformity in nanoparticle. As shown in Fig. 4c by increasing the temperature, PdI reduced and it would be at minimum of 50 °C. On the other hand, lower PdI is observed in the middle range of stirrer rate. Figure 4d represents that by increasing the ratio of HA-DTX to chitosan, PdI would be decreased.

$$ \mathrm{P}\mathrm{d}\mathrm{I}=0.055\hbox{-} 0.041\mathrm{A}\hbox{-} 0.00563\mathrm{B}\hbox{-} 0.00613\mathrm{C}+0.022\mathrm{B}\mathrm{C}+0.02{\mathrm{A}}^2+0.013{\mathrm{B}}^2\hbox{-} 0.014{\mathrm{C}}^2 $$

#### Drug content

Higher amount of drug content is desired and represented an acceptable formulation strategy. HA-DTX to chitosan ratio was the most important factor in drug content. By increasing the ratio of HA-DTX to chitosan, higher drug content would be obtained.

$$ \mathrm{Drug}\ \mathrm{content}=3.16+0.099\mathrm{A}+0.000125\mathrm{C}\hbox{-} 0.018{\mathrm{A}}^2\hbox{-} 0.00138{\mathrm{C}}^2 $$

#### Confirmation of designed optimized experiments

After analyzing data and 3D diagrams by utilizing Box-Behnken method, an optimized formulation achieved which the independent variables were 18.5 for ratio of HA-DTX to chitosan, 723 rpm for stir rate and 50 °C for temperature. It also predicted the amount of dependent factors would be 205 nm for size of nanoparticles, 0.02 for PdI and 3.19 % for DTX content and +21.5 mV for zeta potential. Analysis of variance (ANOVA) and lack of fit parameters for the responses according to quadratic model is provided in Table 3.

**Table 3:** Analysis of variance (ANOVA) and lack of fit parameters for the responses according to quadratic model

Parameters	Source	Sum of squares	Degrees of freedom (df)	Mean squares	F value	*P*-value
Particle Size	Quadratic vs 2FI	15730.13	3	5243.38	4.93	0.0378
Zeta Potential	Quadratic vs 2FI	8.71	3	2.9	5.3	0.0321
PdI	Quadratic vs 2FI	3.11E-03	3	1.04E-03	9.9	0.0065
Drug Loading	Quadratic vs 2FI	1.46E-03	3	4.86E-04	9.99	0.0063
Lack of Fit						
Particle Size	Quadratic	7239.25	3	2413.08	48.26	0.0013
Zeta Potential	Quadratic	2.2	3	0.73	1.79	0.2889
PdI	Quadratic	1.16E-04	3	3.86E-05	0.25	0.8579
Drug Loading	Quadratic	3.39E-04	3	1.13E-04	376.94	0.0001

Three experiments were performed in the lab according to the optimized formulation and there was no significant difference between the obtained and predicted results. The mean amount obtained in these experiments was 234 nm for size of nanoparticles, 0.088 for PdI and 3.18 % for DTX content and +20.03 mV for zeta potential. The mean entrapment efficiency for these experiments was 62.78 %. The entrapment efficiency showed that nanoparticles could be an effective carrier for DTX.

### Characterization of nanoparticles

#### Particle size,distribution and zeta potential

**Fig. 5:**
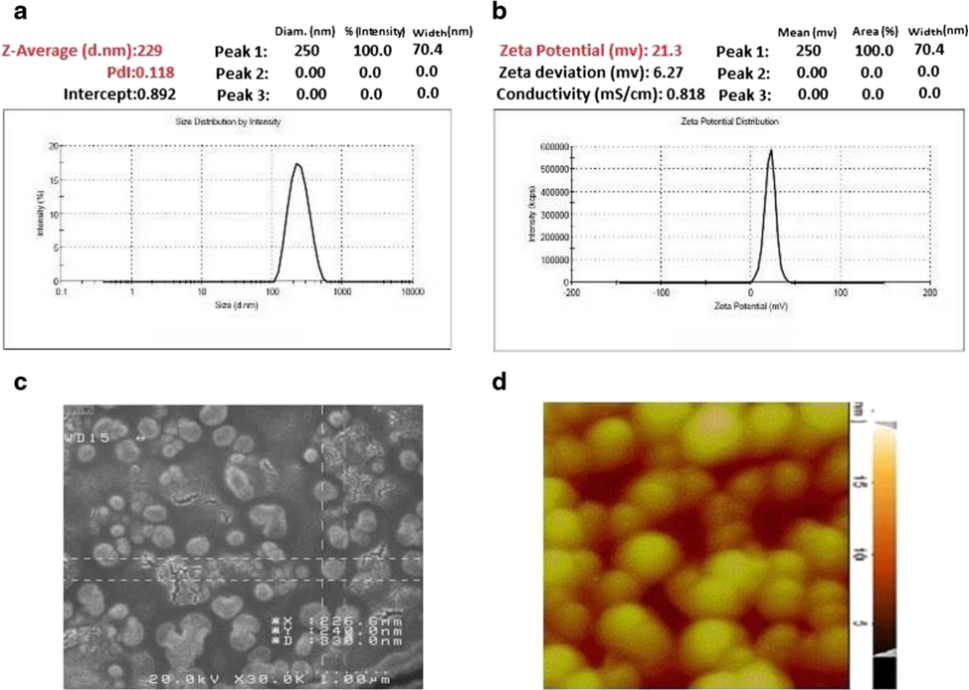
Nanoparticles characterization: **a** particle size and distribution; **b** zeta potential; **c** SEM picture; **d** AFM micrographs

Particle size and size distribution of nanoparticles were measured by dynamic light scattering (DLS). The mean obtained size and PdI of the optimized chitosan coated HA-DTX conjugate nanoparticles were 234 nm and 0.088 respectively (Fig. 5a). Zeta potential of optimized nanoparticles was +20.03 mV which showed complete chitosan coating (Fig. 5b). This amount of zeta potential could provide the appropriate repulsive force to prevent nanoparticle aggregation and improv the stability of formulation.

#### Differential scanning calorimetry

Freeze-dried nanoparticles, conjugated HA-DTX, chitosan, HA, DTX and physical mixture of HA-DTX conjugate and chitosan was analyzed during predetermined increasing temperature rate to obtain DSC thermogram.

**Fig. 6:**
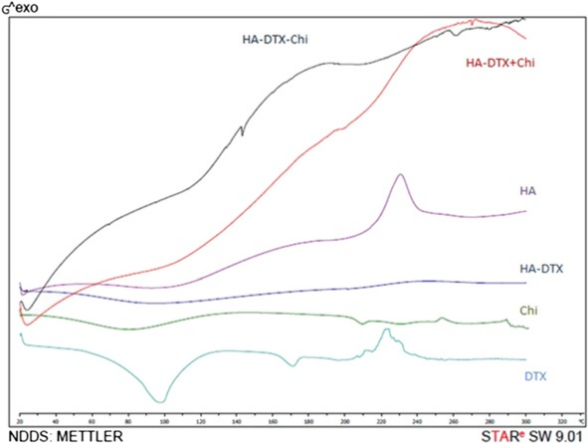
DSC thermogram of freeze-dried nanoparticles, hyaluronic acid-docetaxel (HA-DTX) conjugate, pure chitosan, pure hyaluronic acid (HA), pure docetaxel (DTX) and physical mixture of HA-DTX conjugate and chitosan

The DSC thermograms of DTX exhibit an endothermic peak showed melting around 170 °C. HA exhibited one exothermic peak presenting crystallization around 230 °C. Characteristic peaks of DTX and HA were not exist in the thermograms of conjugated HA-DTX. These findings confirm the development of HA-DTX conjugate (Fig. 6) [32].

DSC thermogram specificities of HA has an influence on DSC thermogram of physical mixture of HA-DTX conjugate and chitosan. Because of the ionic charge of the chitosan (cationic) and HA (anionic), these two compounds can form a charge-transfer bond (the same as PEC formation) which may attenuate the difference in comparing these physical mixture and polyelectrolyte complex. So, DSC thermogram of nanoparticles may not express this influence. These results revealed that chitosan coated nanoparticles protected conjugated HA-DTX and weakened the effect of temperature, which showed the formation of chitosan coated HA-DTX nanoparticles.

#### Nanoparticle morphology

Chitosan coated HA-DTX nanoparticles were morphologically studied by SEM and AFM. SEM showed (Fig. 5c) that nanoparticles are uniform spheres and non-aggregated. AFM micrographs (Fig. 5d) confirmed the spherical shape of nanoparticles too.

### In vitro cytotoxicity

**Fig. 7:**
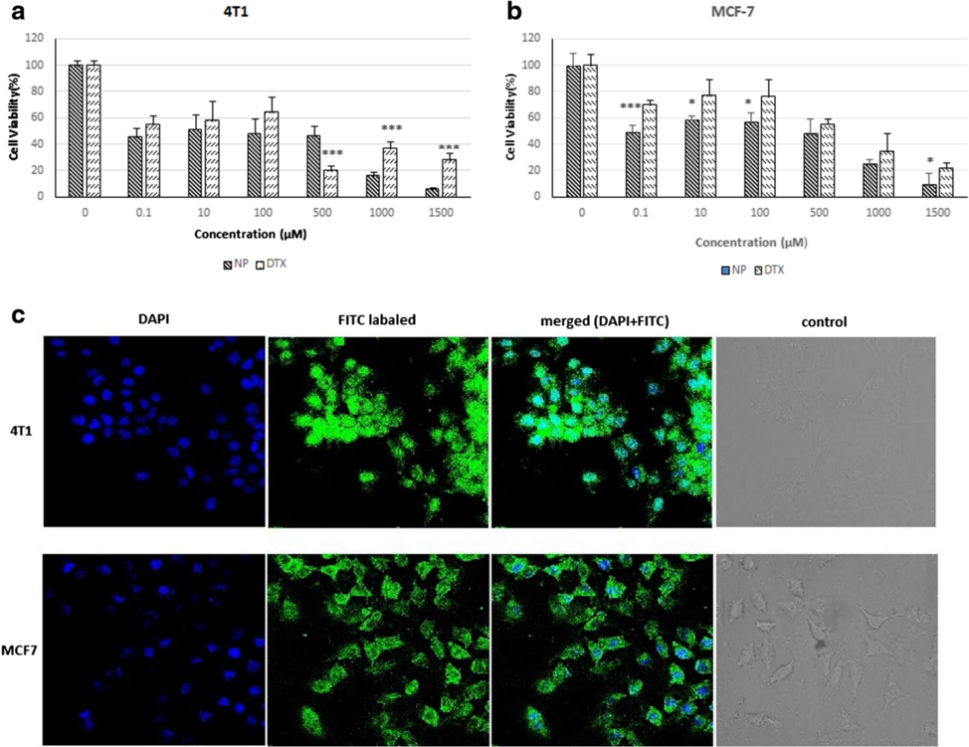
In vitro cell studies: **a** MTT assay of DTX and nanoparticles on 4 T1 cells (blue bar for nanoparticle and red bar for DTX); **b** MTT assay of DTX and nanoparticles on MCF-7 cells (blue bar for nanoparticle and red bar for DTX); **c** uptake image of 4 T1 cell line (nuclear coloring with DAPI, FITC labeled chitosan coated HA-DTX conjugate nanoparticles, merge image of DAPI and FITC labeled chitosan coated HA-DTX conjugate nanoparticles and control cell) and uptake image of MCF7 cell line (nuclear coloring with DAPI, FITC labeled chitosan coated HA-DTX conjugate nanoparticles, merge image of DAPI and FITC labeled chitosan coated HA-DTX conjugate nanoparticles and control cell); *p < 0.05; **p < 0.01; ***p < 0.001

The cytotoxicity of chitosan coated HA-DTX nanoparticles and DTX were assessed by MTT assay on 4 T1 and MCF-7 cell lines. By increasing the amount of nanoparticles or free DTX the cytotoxicity increased. The calculated IC50 of chitosan coated HA-DTX conjugate nanoparticles for 4 T1 and MCF-7 cell lines were 45.34 μM and 354.25 μM respectively while 233.8 μM and 625.9 μM for DTX on 4 T1 and MCF-7 cell lines after 48 h incubation. DTX loaded chitosan nanoparticles were more effective against cancer cells than free DTX drug [30]. Being agree to this result in our study cell viability (%) and IC50 of optimized nanoparticles were less than free drug. It may be because of higher DTX concentration which was available in intracellular space. Small size of nanoparticle as a passive targeting, effect of HA and the adhesive effect of chitosan coat may cause this availability. Therefore, nanoparticles were more potent than free DTX in cytotoxic effect on 4 T1 and MCF-7 cells (Figures 7a, b).

### Cell uptake studies

Entrance of nanoparticles in cancer cells had a direct relation with their observed cytotoxic effect. Free DTX molecules could be transported out by P-glycoprotein (P-gp) pumps, but drug loaded nanoparticles were taken up by cells through an endocytosis pathway. The result represented higher cellular uptake of nanoparticles because of their ability to escape from the effect of P-gp pumps [33]. The uptake of optimized FITC-labeled chitosan-coated HA-DTX conjugate nanoparticles by 4 T1 and MCF-7 after 24 h incubation is shown in Fig. 7c. No treatment cells of each cell line are presented in Fig. 7c as a control. Based on fluorescence intensity, FITC-labeled chitosan-coated HA-DTX nanoparticles showed appropriate entrance into 4 T1 and MCF-7 cells. As a result it can be proposed that developed nanoparticles could bring loaded drug molecules effectively to cell cytoplasm as a novel drug delivery system.

## Conclusion

Water soluble HA-DTX conjugate was prepared according to HA strong affinity for CD44, a cell surface protein which is overexpressed in many cancer cells and cancer stem cells. Chitosan-coated HA-DTX nanoparticles by polyelectrolyte complex method improved DTX availability. The fine optimized formulation was achieved with proper particle size, PdI, zeta potential and drug loading. The biological evaluation of nanoparticles showed they were more potent than free DTX in cytotoxic effect on MCF-7 and 4 T1 cells beside of their appropriate entrance in to cells. These findings need further evaluation to take into account potential improved pharmacokinetic of nanoparticulate drug delivery system.
